# Organizational Wellbeing and Quality of Life in Healthcare Settings: Unexpected Similarities Across Different Roles?

**DOI:** 10.3390/medicina61081437

**Published:** 2025-08-10

**Authors:** Francesco Corallo, Maria Pagano, Anna Anselmo, Irene Cappadona, Davide Cardile, Lilla Bonanno, Giangaetano D’Aleo, Mersia Migliara, Stellario Libro, Smeralda Diandra Anchesi, Rosaria De Luca, Fabio Libro, Antonino Longo Minnolo, Maria Felicita Crupi

**Affiliations:** 1 IRCCS Centro Neurolesi Bonino-Pulejo, Via Palermo, S.S. 113, C.da Casazza, 98124 Messina, Italy; francesco.corallo@irccsme.it (F.C.); davide.cardile@irccsme.it (D.C.);; 2 Azienda Ospedaliera Universitaria Gaetano Martino, Via Consolare Valeria 1, 98124 Messina, Italy; 3 Azienda Ospedaliera Papardo, Contrada Papardo, 98158 Messina, Italy

**Keywords:** organization well-being, healthcare personnel, administrative personnel, professional quality of life

## Abstract

*Background/Objectives*: Occupational well-being and professional quality of life are essential for healthcare sustainability. While clinical staff are presumed to experience higher stress, few studies have compared their experience to that of non-clinical personnel within the same institution. *Methods*: This observational study involved 63 employees from an Italian research hospital: 36 healthcare workers in critical care and 27 administrative staff. Participants completed the Brief Coping Orientation to Problems Experienced Inventory (Brief COPE), an ad hoc organizational questionnaire, and the ProQoL Version 5 (administered to clinical staff only). Non-parametric tests (Mann–Whitney U and Chi-square) were used to explore group differences. *Results*: No significant differences emerged between groups in coping styles or strategies. Significant differences were observed only in reports of work-related injuries (42% of healthcare staff vs. 4% of administrative staff; *p* = 0.002) and perceived disruption caused by vacation requests (64% vs. 26%; *p* = 0.006). Other organizational indicators such as job dissatisfaction, intention to leave, or perceived managerial support did not differ significantly. ProQoL results showed that 53% of healthcare workers had moderate to high burnout, and 47.2% scored high on compassion fatigue, while only 2.7% showed high levels of secondary traumatic stress. *Conclusions*: Despite distinct operational contexts, healthcare and administrative staff reported broadly similar experiences in terms of coping and organizational well-being. These findings challenge assumptions of stark differences across professional roles and suggest that workplace well-being strategies should address the needs of both clinical and non-clinical staff.

## 1. Introduction

Work-related well-being and professional quality of life have emerged as critical dimensions in healthcare systems, where employees are frequently exposed to elevated psychological demands, time pressure, and emotional labor [1]. These stressors are particularly pronounced in acute care settings, such as emergency departments, where workers must make rapid decisions under intense conditions, often with limited resources. Such environments have been consistently linked to adverse outcomes including burnout, compassion fatigue, and impaired job satisfaction [2,3]. Burnout was originally defined by Maslach et al. [4] as comprising emotional exhaustion, depersonalization, and a reduced sense of personal accomplishment, and this remains alarmingly prevalent among healthcare workers. A meta-analysis by Gómez-Urquiza et al. [5] revealed burnout prevalence rates as high as 43% among emergency nurses, with emotional exhaustion as the most affected domain. Similarly, a study of Turkish emergency department personnel found significantly elevated burnout scores across all subscales of the Maslach Burnout Inventory [6]. In Italy, the COVID-19 crisis exposed critical weaknesses in the national health system, including staffing shortages and lack of psychological support. A nationwide study found that nearly half of Italian healthcare workers experienced post-traumatic stress, with elevated rates of depression, anxiety, and insomnia [7]. Frontline staff, especially in emergency and intensive care units, were disproportionately affected, highlighting the urgent need for systemic interventions [8] For instance, a recent umbrella review by Dragioti et al. [9] found that nurses experienced substantial emotional burden during the pandemic, with rates of emotional exhaustion exceeding 48% in some cohorts. These data underscore not only the severity of psychological distress in healthcare workers but also the chronic nature of such conditions, particularly in frontline roles. Moreover, large-scale studies have shown that distress is not equally distributed across roles and departments. Emergency and critical care staff are disproportionately affected, with higher odds of burnout and moral injury compared to colleagues in non-acute settings [10]. In Ireland, for example, Sheehan et al. [11] reported that 74% of emergency healthcare workers met the criteria for moderate to severe burnout during the height of the COVID-19 crisis, regardless of job title. These findings echo results from other European contexts, reinforcing the universality of this phenomenon. Importantly, organizational factors including workload, leadership responsiveness, perceived fairness, and psychosocial safety climate have been identified as both risk and protective factors [12,13]. For instance, Lee & Chang [14] demonstrated that a positive safety climate was inversely correlated with burnout and positively associated with work–life balance across healthcare roles in Taiwan. These results suggest that systemic interventions at the organizational level may buffer the psychological impact of demanding clinical work. In parallel, the concept of resilience has gained prominence in explaining how healthcare professionals adapt to chronic stress and emotionally taxing environments [15]. A qualitative study conducted in the United Arab Emirates found that healthcare and academic staff employ complex interpersonal and intrapersonal strategies ranging from meaning-making to collegial support to maintain psychological resilience [16]. These adaptive processes are essential to mitigating the long-term effects of emotional labor and maintaining workforce sustainability. Research has extensively examined coping strategies and professional quality of life among nurses and physicians, often using instruments such as the ProQOL to evaluate burnout, secondary traumatic stress, and compassion satisfaction [17]. Studies also highlight how individual and organizational resources including perceived support, autonomy, and mental health resources mediate these outcomes across contexts [18,19]. However, most of the existing literature focuses on clinical personnel; less is known about how such phenomena compare to administrative staff within the same institutional environment [20]. To address this gap, the current study compares two populations within IRCCS Neurolesi Bonino-Pulejo institute, a public structure with proven expertise in neurological and neurorehabilitative medicine that is set in the Italian context. By contrasting these groups, we aim to examine organizational well-being, quality of working life, and coping strategies, and to explore whether frontline exposure correlates with more adverse outcomes. We aim to (a) evaluate and compare organizational well-being and perceived quality of working life between ward and administrative staff; (b) describe coping strategy profiles in each group via Brief COPE; and (c) characterize professional quality of life (burnout, compassion fatigue, and compassion satisfaction) in healthcare personnel using ProQOL Version 5.

## 2. Materials and Methods

This is an observational study with a total duration of one year, determined by the participation of the enrolled subjects through the completion of administered questionnaires, followed by data analysis and publication of the results. The study was conducted in accordance with the principles outlined in the Declaration of Helsinki; however, based on the type of study and in accordance with Italian law, a preliminary evaluation by an ethics committee was not deemed necessary. Indeed, since no individual patient data were reported, only aggregate data provided in the form of responses from the organization’s practitioners, ethics committee approval was not required. It should also be noted that this is a non-randomized study, and sampling was based on participant availability at the time of data collection.

Questionnaires concerning the organizational well-being experienced within the hospital were administered to all individuals deemed eligible to participate in the study. The analysis was performed at the level of individual items.

**Figure 1:**
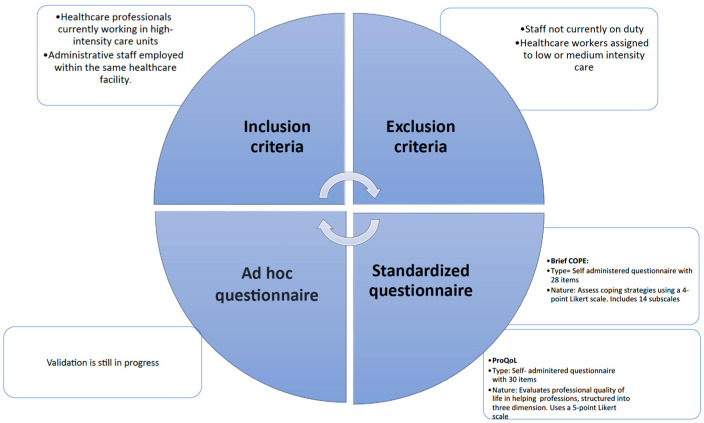
Procedure of the study.

The study procedure is illustrated in the flowchart shown in Figure 1.

### 2.1. Stage 1: Enrollment

During the survey, 213 questionnaires sent via a broadcast list were distributed to all administrative staff and all staff in critical wards of the hospital; specifically, the questionnaires were sent to 43 administrative staff and 170 healthcare staff belonging to critical units. The anonymized data of each participant were shared in a database, which was accessible to the study collaborators through previously assigned login credentials

### 2.2. Stage 2: Administration

Subsequently, in the second phase, standardized tests were administered to assess coping strategies, along with an ad hoc questionnaire for organizational analysis and, for healthcare personnel only, a tool for evaluating professional quality of life.

These tests included the following:

- The Brief Coping Orientation to Problems Experienced Inventory (Brief COPE): is a self-report instrument developed by Charles S. Carver in 1997 [21], designed to evaluate the coping strategies individuals use in response to stress.
- Structure: A total of 28 items, grouped into 14 subscales (e.g., active coping, denial, substance use, and use of emotional support).
- Scoring: Each item is rated on a 4-point Likert scale (1 = “I haven’t been doing this at all” to 4 = “I’ve been doing this a lot”). This tool does not yield a total score but the use of the individual subscales is interpreted independently.
- Interpretation: Scores for each subscale are calculated by summing the responses to the two items associated with that subscale; higher scores indicate greater use of that specific coping strategy.
- Reliability: Reported Cronbach’s alpha coefficients for the subscales typically range from 0.50 to 0.90, depending on the population and subscale.
- Ad hoc Questionnaire differentiated by sectors to assess operational and structural challenges, aimed at promoting work well-being hindered by underestimated mechanisms.
- ProQoL [Professional Quality of Life]: ProQoL was developed by Beth Stamm in 2005 [17] to assess the professional quality of life among individuals in helping professions.
- Structure: A total of 30 items, divided into three subscales: Compassion Satisfaction, Burnout, and Secondary Traumatic Stress.
- Scoring: Items are rated on a 5-point Likert scale (1 = “Never” to 5 = “Very often”), with 10 items per subscale. Scores for each subscale are calculated by summing the responses and classifying them into three ranges, respectively, Low (≤22), Medium (23–41), or High (≥42).
- Interpretation: Each subscale score is calculated by summing relevant items. Higher Compassion Satisfaction scores indicate fulfillment in the caregiving role; higher Burnout or Secondary Traumatic Stress scores indicate emotional strain.
- Reliability: Internal consistency values reported in the literature include Cronbach’s alpha of approximately 0.88 (CS), 0.75 (BO), and 0.81 (STS).

Feasibility was assessed by examining participants’ adherence to the monitoring program, measured through the completion rate of the questionnaires administered at the enrollment stage. Specifically, the following aspects were considered: the percentage of participants who fully completed the required instruments, the average time taken to complete them, and the perceived clarity of the items.

### 2.3. Stage 3: Analysis

A nonparametric analysis was carried out because the results of the Shapiro normality test indicated that most of the target variables were not normally distributed. Numerical data are presented as the median and interquartile range (first and third quartiles) for non-normally distributed variables. The Chi-square test and the Mann–Whitney U test were used for inter-group comparisons, as appropriate. Specifically, the Chi-square test was used to evaluate whether there was a significant association between the groups (Healthcare workers or Administrative staff). Before performing the Chi-square test, expected frequencies were examined to ensure that the assumptions of the test were met (no cell with expected frequency < 1 and not more than 20% of cells with expected frequency < 5). For each significant Chi-square test, effect sizes were computed using Cramér’s V, and for Mann–Whitney U tests; the effect size *r* was calculated $r=\left(Z/\sqrt{N}\right)$.

This additional reporting improves the interpretability of group differences. Analyses were performed using an open source R3.0 software package. A 95% of confidence level was set with a 5% alpha error. Statistical significance was set at *p* < 0.05.

## 3. Results

### 3.1. Sociodemographic Results

The overall response rate was 29.58%. Specifically, twenty-seven responses were collected from administrative staff, corresponding to a response rate of 62.79%, while thirty-six responses were obtained from healthcare personnel working in critical care areas, reflecting a response rate of 21.17%. The sociodemographic characteristics of the participants are presented in Table 1.

### 3.2. Analysis

A Chi-square test revealed a significant association between the group (Healthcare workers or Administrative staff) and the frequency of workplace accidents (ꭓ2 = 9.82, df = 1, *p* = 0.002, Cramer’s V = 0.39). Similarly, a significant association was found in the question regarding whether the request for vacation days caused organizational disruption for the hospital (ꭓ2 = 7.46, df = 1, *p* = 0.006, Cramer’s V = 0.34). No significant associations were observed between the group and several other variables. These include the frequency of falling ill more than three times in the past year due to work-related physical or psychological reasons (ꭓ2 = 0.004, df = 1, *p* = 0.95, Cramer’s V = 0.008), the presence of unused vacation days from the previous year (ꭓ2 = 0.72, df = 1, *p* = 0.40, Cramer’s V = 0.11), and having considered the possibility of changing jobs (ꭓ2 = 0.03, df = 1, *p* = 0.85, Cramer’s V = 0.02). Additionally, no significant relationships emerged concerning experiencing verbal and/or physical assault from users (ꭓ2 = 2.50, df = 1, *p* = 0.11, Cramer’s V = 0.20), the perception of whether skills have been valued by the hospital (ꭓ2 = 0.66, df = 1, *p* = 0.42, Cramer’s V = 0.10), expressing work-related difficulties to superiors (ꭓ2 = 0.002, df = 1, *p* = 0.96, Cramer’s V = 0.005), and making proposals for improvements or solutions (ꭓ2 = 1.72, df = 1, *p* = 0.19, Cramer’s V = 0.19). Furthermore, there is no significant association between the group and receiving comprehensive information regarding their requests (ꭓ2 = 0, df = 1, *p* = 1, Cramer’s V = 0), as the data suggest that both groups received information at the same rate. Table 2 shows the details of the questions in the ad hoc questionnaire and the response rate by group.

**Figure 2:**
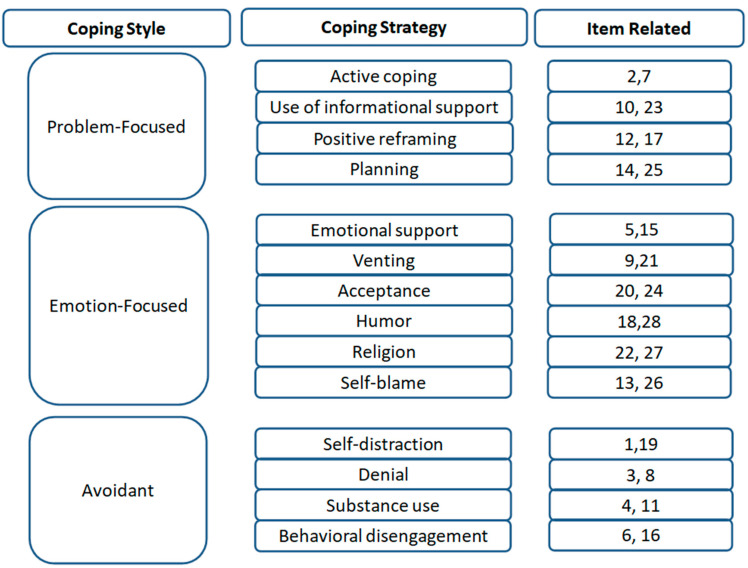
Structure of Brief Cope.

Based on the structure of the test, data were aggregated on three levels of analysis: coping styles, coping strategies, and individual items (Figure 2).

U-Mann–Whitney showed no significant difference between groups (Table 3). No significant differences were observed between groups in coping style and coping strategies.

### 3.3. Descriptive Analysis of ProQoL Results

Descriptive statistics were used to summarize the scores of participants in the “healthcare workers” group for each subscale of the ProQoL. Participants’ raw scores on each ProQOL subscale were categorized into Low (≤22), Average (23–41), and High (≥42) ranges. Absolute frequencies per category were converted into relative frequencies (percentages).

**Figure 3:**
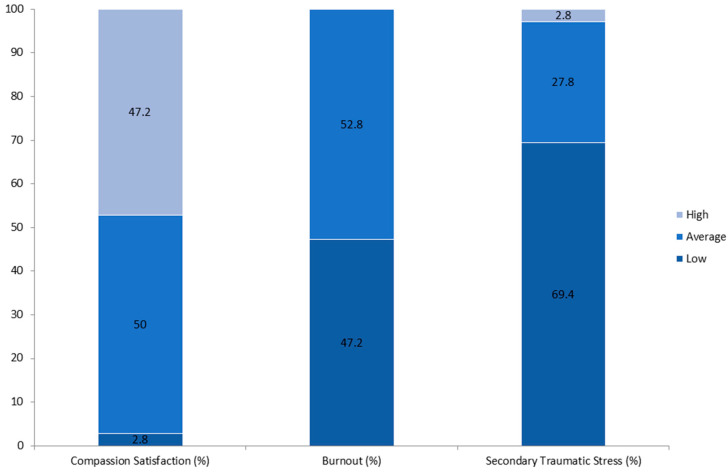
Healthcare workers’ ProQoL subscale scores (Compassion Satisfaction, Burnout, Secondary Traumatic Stress) by risk level (low, average, high) among healthcare workers (N = 36).

The results of ProQoL (Figure 3) administered to healthcare workers only showed that about 53% of professionals scored at risk for the “burnout” area and the remaining 47% scored low risk. For the “secondary stress trauma” area, the scores indicated a low percentage for high risk (2.7 percent), 27.8 percent for medium risk, and 69.4 percent for low risk. For the “compassion fatigue” section, 50 percent of professionals scored medium risk, 2.8 percent low risk, and 47.2 percent high risk.

## 4. Discussion

The previous literature highlights that healthcare workers’ dissatisfaction may lead to significant economic and social costs due to its negative impact on the work environment, performance, and quality of healthcare services provided [21].

The overall response rate was low, with particularly limited participation among healthcare workers. While previous research has linked low response rates to sociodemographic factors [22], other potential explanations include time constraints or a perception that organizational surveys are of low priority. A recent study highlights that healthcare professionals often experience a mismatch between high job demands and limited available resources, identifying this as a key system-level work stressor [23]. This imbalance may prompt healthcare workers to reassess their work priorities in a way that discourages participation in organizational or non-clinical surveys. In addition, the low response rate may also reflect a lack of trust in the organization or a limited recognition of the importance contributing to quality improvement efforts, processes that require active participation from all stakeholders [24]. Future studies comparing healthcare workers’ engagement in clinical versus organizational initiatives could help uncover additional, yet unidentified, factors influencing survey participation and involvement in non-clinical activities.

In this study, we explored organizational well-being, coping strategies, and professional quality of life among healthcare and administrative staff within the same institution. Contrary to our initial hypothesis, no significant differences emerged in coping strategies between the two groups. This finding is in line with previous studies suggesting that professional role may not be the main determinant of coping styles, which are often shaped by broader institutional culture and environmental stressors [21,25].

Significant differences were observed only in work-related injuries and in the perception that vacation requests caused organizational disruption, both more frequent among healthcare workers. These results are consistent with the literature, highlighting the unique operational burdens faced by clinical staff, including physical risk and rigid shift structures [26]. However, the lack of significant differences in key indicators such as perceived support, job satisfaction, sick leave, and intention to leave, suggests that administrative personnel may experience comparable levels of structural and psychosocial strain [27,28].

The ProQoL results further support this interpretation: A total of 53% of healthcare workers showed moderate to high levels of burnout, and 47.2% scored high in compassion fatigue figures consistent with international data on emergency nurses [5,29]. Interestingly, the prevalence of high secondary traumatic stress was low (2.7%), possibly reflecting episodic rather than chronic exposure to traumatic content.

The absence of group differences in Brief COPE scores echoes findings from other studies showing that both clinical and non-clinical workers tend to employ similar coping strategies, particularly emotion-focused and problem-solving techniques, when facing institutional stress [30,31]. Maladaptive coping (e.g., substance use, denial) remained low across groups, which may indicate the presence of internal or external resilience factors [15,32,33].

These results are consistent with the Job Demands-Resources model (JD-R) and the Psychosocial Safety Climate (PSC) framework, which emphasize that well-being is more influenced by systemic and contextual factors (e.g., leadership, perceived fairness, workload) than by occupational role alone [12,13,14,34,35].

### 4.1. Limitations

This study highlights an important issue affecting all workers in helping professions, such as those in healthcare, by including both clinical and administrative staff. However, several limitations must be acknowledged.

First, the cross-sectional design limits the ability to establish causal relationships between the variables examined. While associations can be observed, directionality remains speculative. Future longitudinal research is necessary to assess how these relationships evolve over time.

Second, the sampling method was based on voluntary participation and convenience sampling, which may have introduced self-selection bias. Participants who chose to complete the questionnaire may differ systematically from those who did not, in terms of psychological characteristics or workplace experiences.

Additionally, the study was conducted in a single-center setting and included only healthcare workers from critical care units and administrative staff, which limits the generalizability of the findings. These results may not reflect the broader institutional environment or the experiences of staff working in other departments.

Another limitation relates to the use of validated tools not specifically designed for administrative staff, which could have affected the comparability between the two groups. Future studies could benefit from using occupation-specific quality of life assessment tools tailored to administrative personnel, to increase the precision and relevance of the results.

### 4.2. Future Perspectives

To address these limitations, future research should aim to include a larger and more diverse sample, extending the investigation to staff from other departments and non-clinical roles. This would enhance the representativeness of the data and support broader and more generalizable conclusions regarding occupational well-being in healthcare settings.

Moreover, adopting a longitudinal design would allow researchers to monitor changes over time in professional quality of life, stress perception, and coping strategies, offering a more dynamic and causal understanding of these factors.

It would also be beneficial to explore the use of occupation-specific tools for different staff categories. For example, while the ProQoL was administered only to healthcare professionals in the current study due to its validation for helping professions, future studies may consider developing or adopting tailored instruments for administrative personnel. This would allow for a more precise and equitable assessment of work-related well-being across the entire workforce.

### 4.3. Policy Recommendations for Organizational Improvement

Based on the study findings, the following policies are recommended to improve organizational climate and staff well-being:

- Implement structured psychological support programs, particularly for staff in high-stress environments.
- Promote a culture of safety and organizational well-being, encouraging open communication and collaboration between clinical and administrative sectors.
- Provide targeted training on stress management and coping strategies, adapted to the specific roles and responsibilities of different staff categories.
- Regularly assess organizational climate and perceived workload, using validated tools to identify emerging risks and intervene proactively.

## 5. Conclusions

Organizational well-being in health care companies is something that needs to be cultivated and disseminated. Interest should include both workers in direct contact with users and those affected by back-office activities. In the future, the analysis conducted could be further expanded by involving other health care workers, implementing a comparison of health care workers subjected to different sources of stress such as long-term care, rehabilitation, and psychiatry. It would be interesting to make a comparison with operators of outpatient one-day services who have contact with larger but faster volumes of users. It is essential for managers in health care facilities to reflect on how to promote a positive organizational climate that can generate favorable emotions in employees, reduce job stress, and improve their involvement.

## Figures and Tables

**Table 1:** Sociodemographic characteristics.

	Healthcare Workers n (%)	Administrative Staff n (%)	Chi Square	*p*-Value (Cramer’s V)
Gender				
Male	15 (42%)	14 (52%)	0.30	0.58 (V = 0.07)
Female	21 (58%)	13 (48%)		
Age				
<30 years	1 (3%)	0	3.75	0.15 (V = 0.24)
30–50 years	17 (47%)	19 (70%)		
>50 years	18 (50%)	8 (30%)		
Marital status				
Married/Cohabiting	25 (69%)	19 (70%)	0	1 (V = 0)
Single/Widowed	11 (31%)	8 (30%)		
Education level				
Junior college degree	6 (17%)	11 (41%)	7.74	0.02 * (0.35)
Bachelor’s degree	9 (25%)	1 (4%)		
Master’s degree and other specializations	21 (58%)	15 (56%)		
Contract Position				
Fixed-term	1 (2%)	3 (11%)	1.87	0.39 (0.17)
Permanent	33 (92%)	23 (85%)		
Other	2 (6%)	1 (4%)		

* *p* < 0.05.

**Table 2:** Inter-group comparison of responses to the ad hoc questionnaire.

Item	Healthcare Workers (N = 36)		Administrative Staff (N = 27)		Notes
	% Yes	% No	% Yes	% No	
Have you had any work-related injuries?	42	58	4	96	*p*-value = 0.002
Have you been sick frequently in the past year (greater than three episodes) for work-related physical or psychological reasons?	11	89	7	93	non-significant comparison
Do you have any unused vacation days left over from the previous year?	89	11	78	22	non-significant comparison
Has your request for vacation days resulted in organizational discomfort for the hospital company?	64	36	26	74	*p*-value = 0.006
Have you ever thought about the possibility of changing jobs?	50	50	56	44	non-significant comparison
Have you ever been in situations where you were verbally and/or physically assaulted by users?	53	47	30	70	non-significant comparison
Do you feel that your skills have been valued by the hospital company?	39	61	26	74	non-significant comparison
Have you ever expressed any difficulties in the work environment to your superiors?	78	22	74	26	non-significant comparison
Have you made any improvement or solution proposals?	93	7	75	25	non-significant comparison
Have you received comprehensive information regarding any of your requests?	33	67	33	67	non-significant comparison

**Table 3:** U-Mann–Whitney Results of Brief COPE (N = 63).

Item	Healthcare Workers Median (I–III Quartile)	Administrative Staff Median (I–III Quartile)	*p*-Value	Effect Size
1. I’ve been turning to work or other activities to take my mind off things	2.0 (2.0–3.0)	1.0 (1.0–2.5)	0.10	r = 0.21
2. I’ve been concentrating my efforts on doing something about the situation I’m in	2.0 (2.0–3.0)	3.0 (2.0–4.0)	0.33	r = 0.12
3. I’ve been saying to myself “this isn’t real.”	2.0 (1.0–2.0)	2.0 (1.0–2.5)	0.56	r = 0.08
4. I’ve been using addictive behaviors or substances to make myself feel better	1.0 (1.0–1.0)	1.0 (1.0–1.0)	0.75	r = 0.04
5. I’ve been getting emotional support from others	2.0 (2.0–2.0)	2.0 (1.5–2.0)	0.97	r = 0.01
6. I’ve been giving up trying to deal with it	1.0 (1.0–2.0)	1.0 (1.0–2.0)	0.92	r = 0.01
7. I’ve been taking action to try to make the situation better	3.0 (3.0–3.0)	3.0 (2.0–3.0)	0.47	r = 0.09
8. I’ve been refusing to believe that it has happened	1.0 (1.5–2.0)	1.0 (1.0–2.0)	0.94	r = 0.01
9. I’ve been saying things to let my unpleasant feelings escape	2.0 (1.0–2.0)	2.0 (1.0–2.5)	1.0	r = 0.00
10. I’ve been getting help and advice from other people	2.0 (2.0–2.0)	2.0 (2.0–2.0)	0.96	r = 0.01
11. I’ve been using alcohol or other drugs to help me get through it	1.0 (1.0–1.0)	1.0 (1.0–1.0)	0.85	r = 0.03
12. I’ve been trying to see it in a different light, to make it seem more positive	2.0(2.0–3.0)	2.0 (2.0–3.0)	0.90	r = 0.02
13. I’ve been criticizing myself	2.0 (1.0–2.0)	1.0 (1.0–3.0)	0.97	r = 0.01
14. I’ve been trying to come up with a strategy about what to do	3.0 (2.0–3.0)	2.0 (2.0–3.0)	0.25	r = 0.15
15. I’ve been getting comfort and understanding from someone.	2.0 (1.75–2.0)	2.0 (1.0–2.0)	0.66	r = 0.06
16. I’ve been giving up the attempt to cope	1.0 (1.0–1.0)	1.0 (1.0–1.0)	0.50	r = 0.09
17. I’ve been looking for something good in what is happening	3.0 (2.0–3.0)	2.0 (2.0–3.0)	0.86	r = 0.02
18. I’ve been making jokes about it.	2.0 (2.0–3.0)	2.0 (1.0–3.0)	0.32	r = 0.13
19. I’ve been doing something to think about it less, such as going to movies, watching TV, reading, daydreaming, sleeping, or shopping	2.0 (2.0–3.0)	2.0 (1.5–3.0)	0.49	r = 0.09
20. I’ve been accepting the reality of the fact that it has happened	2.0 (2.0–3.0)	2.0 (2.0–3.0)	0.85	r = 0.03
21. I’ve been expressing my negative feelings	2.0 (2.0–3.0)	2.0 (1.0–3.0)	0.70	r = 0.05
22. I’ve been trying to find comfort in my religion or spiritual beliefs	2.0 (2.0–3.0)	2.0 (1.0–2.0)	0.22	r = 0.16
23. I’ve been trying to get advice or help from other people about what to do	2.0 (2.0–2.0)	2.0 (2.0–2.0)	0.89	r = 0.02
24. I’ve been learning to live with it.	2.0 (2.0–3.0)	2.0 (2.0–3.0)	0.51	r = 0.08
25. I’ve been thinking hard about what steps to take.	2.0 (2.0–3.0)	3.0 (2.0–3.0)	1.0	r = 0.001
26. I’ve been blaming myself for things that happened	2.0 (1.0–2.0)	2.0 (1.0–2.0)	0.86	r = 0.02
27. I’ve been praying or meditating	2.0 (2.0–3.0)	2.0 (1.0–2.0)	0.14	r = 0.19
28. I’ve been making fun of the situation	2.0 (1.75–2.0)	2.0 (1.0–2.0)	0.72	r = 0.05

## Data Availability

Datasets are available to download on request. Requests should be directed to the corresponding author.
